# The Yearlong Effect of COVID-19 on Food Safety: Consumer Practices and Perceptions Using Longitudinal Consumer Surveys and Focus Groups

**DOI:** 10.3390/foods14040551

**Published:** 2025-02-07

**Authors:** Merlyn Suzanne Thomas, Elma Kontor-Manu, Yaohua Feng

**Affiliations:** Department of Food Science, Purdue University, West Lafayette, IN 47906, USA

**Keywords:** food safety practice, COVID-19 pandemic, handwashing, perception, foodborne illness

## Abstract

Coronavirus disease 2019 (COVID-19) caused many consumers in the United States to change their perceptions and food handling practices at the height of the pandemic. We used a quantitative–qualitative mixed-method approach to assess consumers’ risk perceptions and food safety practices during the COVID-19 pandemic. Nine waves of surveys were distributed to an online consumer panel over a 13-month period (April 2020–May 2021), and four waves of focus groups were conducted (May–July 2020 and June 2021). While the pandemic elevated peoples’ perceptions of risks related to food safety practices, many consumers were reverting to past behaviors by May 2021. Participants asserted high confidence in their food safety measures; however, they perceived a low risk of contracting COVID-19 from food. Contrasts in food handling became apparent when assessing different age groups; observations revealed that practices in households with high-risk individuals differed significantly from those without. Although not recommended, the practice of washing produce with soap was consistent, predicting a possible continuation of this practice over time. This study highlights various factors that food safety educators and policymakers need to consider for effective communication about risks associated with food safety practices in preparation for pandemics and other major health events.

## 1. Introduction

Foodborne illness is not a new topic, and while advancements are continuously being made to further mitigate the risks, consumers can use established methods to protect themselves [1]. However, major societal changes and shifts in living conditions can create fluctuations in perceptions of risks and induce resultant changes in practices. Behavior models have shown that behavioral changes in health-related situations correspond to varying levels of risk perception [2]. Due to perceptions of increased risk during major public health events, pandemics may trigger consumers to become hyperaware of their general practices, including those related to food handling [3]. The coronavirus disease 2019 (COVID-19) pandemic was a major public health event that affected consumers’ food handling practices. Although the main mode of transmission of the SARS-CoV-2 virus is human-to-human contact through respiratory droplets and contact routes [4], some studies have reported other indirect routes of transmission, including the deposition of the virus on inanimate surfaces that could cause infection when hands touch those surfaces and then touch the eyes, nose, or mouth [5,6]. SARS-CoV-2 has been reported to be stable and viable on various surfaces and under diverse environmental conditions [7,8]. The survival of the virus on various categories of foods was investigated by Jia and colleagues [9]. They reported the persistence of the virus on meat and deli foods for three weeks under 4 °C refrigeration conditions. Studies such as these reiterate the importance of food handling practices, including washing hands, cleaning contact surfaces, and rinsing produce to prevent probable infection via indirect routes.

In the United States, COVID-19 changed the perceptions and food handling habits of many consumers who sought to protect themselves from contracting SARS-CoV-2 [10,11]. While many of these changes were productive, such as washing hands with soap, some were undesirable, like washing fruits and vegetables with soap and water [11]. Public health officials must understand the motivations for certain food handling practices, because consumers may or may not retain these practices after a pandemic or other emergent health problems. The practices that consumers adopt can affect not only themselves but also those around them, including individuals who may be at a higher risk of disease [12].

Previous longitudinal studies have assessed consumer behaviors and perceptions during past pandemics [13,14]. These studies helped in formulating effective risk communication during those periods. Some current studies have investigated consumers’ food safety behaviors and perceptions during the COVID-19 pandemic. A study by Min and colleagues [15] found that consumers who focused on COVID-19-related issues had higher food safety knowledge and employed safer food handling practices. This finding demonstrated the importance of safety information dissemination in shaping consumers’ perceptions of risk and in influencing their practices. Mucinhato and colleagues [16] also reported how knowledge and risk perception contributed to increased safety in food handling practices during the pandemic. Other studies have shown attitudes, norms, and perceived behavioral control as predictors in consumers’ intention to apply safe food handling methods [17].

Although some studies have investigated food safety practices and risk perceptions during the COVID-19 pandemic, at the time of writing this paper, most of these studies have been conducted on populations outside the United States. In addition, limited information is available on the observable trends of these food safety practices over an extended period. We therefore conducted this study using a longitudinal approach to assess U.S. consumers’ food safety practices during the COVID-19 pandemic. The goal was to collect information over a period of time to discern any trends in practices that could potentially be helpful information when developing food safety risk communications. Such information is important because understanding the target audience is key in developing effective risk communication strategies. Monitoring the changes in safe food handling practices among U.S. consumers is necessary to illuminate their practices and determine predictors that potentially affect the continual adaptation of these food handling practices during public health events. We conducted our online longitudinal study over 13 months in the pandemic using nine waves of surveys in combination with four waves of focus groups.

The qualitative data that we collected from the online focus groups help to explain the quantitative survey data. Focus groups allow people to express their thoughts and opinions about an issue, product, or service through a facilitated discussion [18]. The use of a quantitative–qualitative mixed-methods approach can increase confidence in results and not only further explain what is happening but also postulate on why something is happening. Previous studies have utilized this mixed-methods approach to assess consumer food safety perceptions and behaviors [19,20]. The objective of this study was to assess the ways in which the COVID-19 pandemic influenced food handling practices and food safety perceptions among U.S. consumers over 13 months in the pandemic. This information can help policymakers and food safety educators understand consumers’ reactions and responses to food safety initiatives and apply those insights in the development of effective risk communication messages in the event of another major health event.

## 2. Materials and Methods

The Institutional Review Board (IRB) approved our research protocols before data collection began (IRB # 2020-558). This study used both quantitative (online surveys) and qualitative (online focus groups) approaches to explore food safety perceptions and behavior changes among U.S. consumers. This study collected and analyzed data to identify any changes over time.

### 2.1. Survey Study Procedures

To assess changes for 13 months, surveys were distributed in nine waves [10,11]. The waves were scheduled once per month from April to August 2020 and in October 2020, January 2021, March 2021, and May 2021. While the researchers did not gather data from the same participants each month, participants were recruited from the same pool for each wave, creating the likelihood that individual participants may have been involved in the survey process throughout the year. At least 700 respondents were recruited each month, with survey management and distribution performed using Qualtrics XM. This sample size and our collection method are similar to those of a previous study assessing risk perceptions related to avian influenza in 2006–2007 [14].

Survey respondents met three inclusion criteria: they had to be primary food preparers, primary grocery shoppers, and at least 18 years old. Demographic screening questions regarding age, gender, income, and other characteristics were included to match the study population to the general U.S. population [21]. Additional demographic questions were asked to gauge the effect of household conditions on food safety practices and perceptions. For example, respondents were asked if anyone in their household would be considered at high risk for foodborne illness [12].

The survey design was based on previous studies related to pandemics and food safety [14,22,23]. Survey items included topics related to food handling practices and perceptions during the COVID-19 pandemic. To detect the respondents’ level of disengagement, two survey items contained the wording “if you are paying attention, please do not select this option”. Phrases such as this are known as “instructional manipulation checks” (IMCs). This study used IMCs as an additional screening component to improve the quality of the information gathered, because past studies have shown that online survey respondents do not necessarily pay attention when completing surveys [24]. Prior to its launch, the survey was pilot-tested among 26 consumers for face validity. Along with that, Cronbach’s alpha test was conducted to assess internal consistency among the various scales within the survey; the alpha ranged from 0.65 to 0.91. This range is considered acceptable since an ideal alpha coefficient is 0.7 or higher, depending on the scale [25].

The survey data were analyzed using SPSS Statistics 26.0 (IBM Corp., Armonk, NY, USA). Analysis was performed for descriptive data, for data within each month, and for longitudinal data across the months. Because a paired-sample *t*-test can assess significant differences between matched pairs of data [26], we used a paired-sample *t*-test in this study to compare food safety vs. COVID-19 perceptions within each month. In order to compare levels of food handling practices against demographic data within each month in this study, we used Welch’s *t*-test, which is appropriate for comparing the means of two independent groups among which variance is unequal [27]. We analyzed longitudinal data using one-way ANOVA and Games–Howell post hoc tests as a means to assess differences in the levels of practices across the months with and without demographic factors taken into consideration [28].

### 2.2. Focus Group Study Procedures

Survey data provide information on what is occurring, while focus group discussions among participants who engage in a discussion using open-ended questions aid in explaining these occurrences [18]. Previous studies related to food safety and consumer behavior have utilized this method to gather insight [20,29]. This present study used two sets of focus group discussions; one set was conducted earlier in the pandemic as a longitudinal study in May, June, and July in 2020 (2020 sessions), and the second set was conducted in June 2021 (2021 sessions) to gauge changes in consumer behavior and collect additional insight that could explain changes in the present study’s survey data. The original focus group script also was pilot-tested among a convenient sample of consumers. The pilot tests gave researchers insight that helped them create probing questions designed to evoke further thoughts and discussion from the participants [18].

Participants for the 2020 sessions were recruited from the April 2020 wave of surveys, and the sessions were conducted online via Webex version 40.2.14.19 (Cisco Systems Inc., San Jose, CA, USA). Volunteers for this study were split into groups—low, medium, or high—based on the number of COVID-19 cases in their state of residence (by 27 April 2020). Convenience samples of up to 12 participants were recruited for each group. Because there was a smaller number of participants in the low- and medium-COVID-19 groups, the researchers combined them into one group [10,11]. The participants for the 2021 sessions were recruited from the May 2021 wave of surveys, and the sessions were conducted using Zoom video communications (version 5.7.6). Online video chatting enabled communication with participants in various states throughout the nation while maintaining social distancing during the pandemic [30,31]. During the sessions, which lasted a maximum of 90 min each, a moderator asked questions and a co-moderator took notes. After each session, the moderator and co-moderator discussed important highlights from the session, which aided in subsequent data analysis.

All recruitment was voluntary, and the researchers used purposeful or purposive sampling as prescribed to maximize the quality of information from participants in order to fulfill our objective [32]. Volunteers for the focus groups were contacted and placed into groups based on the times and dates that they were available to meet. Because they were recruited from the pool of survey respondents, all focus group participants met the same inclusion criteria: primary meal preparers, primary grocery shoppers, and at least 18 years old.

The focus group script consisted of questions related to the COVID-19 pandemic and was separated into sections based on three topics: preventive measures, food safety concerns, and food safety information. Preventive measures included questions about the practices that participants engaged in to protect themselves from contracting COVID-19 and their thoughts on the effectiveness of these practices. Food safety concerns included questions about participants’ food safety practices and food purchasing habits and their plans for continuing their food handling practices after the pandemic was over. To create a baseline understanding of how the participants felt toward food safety prior to the pandemic, the researchers also asked if the participants had been aware and/or cautious of foodborne illness prior to the COVID-19 pandemic. The last section assessed the food safety information that participants had received during the pandemic, the sources they trusted for this type of information, and their preferred delivery format.

The focus group discussions were audio- or video-recorded and transcribed word-for-word by one researcher, after which another researcher validated the accuracy of the transcriptions. The transcriptions were uploaded to NVivo version 12 (QSR International) to be coded and analyzed using thematic analysis [33]. For this study, data from the 2020 sessions were compared to those from the 2021 sessions to explain changes in behavior and attitudes. To analyze the 2020 sessions, one researcher (R1) independently coded the transcripts from the first month using a deductive and inductive approach. To reduce bias in codebook development, another researcher (R2) independently reviewed and coded two transcripts using the codebook. Both researchers discussed the codebook and came to a consensus. Because this study was longitudinal, other sessions yielded other codes that were added to the codebook. Both researchers came to a consensus to finalize the codebook after the sessions.

The transcriptions for the 2021 sessions were initially reviewed and analyzed by the researcher R1 using an established codebook that the same two researchers (R1 and R2) previously created for the first set of focus group discussions (2020 sessions). Along with the deductive approach of using an already-established codebook, R1 also used an inductive approach and added six additional codes that classified noteworthy responses from the participants [34]. After R1 coded all the data, R2 verified the accuracy of the additions to the codebook and the transcriptions that were added under each code. The two researchers worked together to gain consensus and reduce bias. From these codes, both researchers selected a few that explained the data found in the survey and organized the codes into themes. Table 1 identifies the selected codes (15), themes (3), and representative participant codes.

## 3. Results

### 3.1. Participant Demographics

Table S1 includes all survey respondent information and demographics tabulated by month. A total of 6496 respondents (700+ respondents per month) completed the survey. The survey was quota-controlled for participants’ sociodemographic characteristics to be nationally representative. The study demographics closely matched the US Census demographics with slight changes seen in fluctuating numbers within the months for educational level. In July 2020, October 2020, and January 2021, a slight overrepresentation of higher-educated individuals was reported. In all months, 37–45% of respondents were living in a household with at least one high-risk individual, including older adults (65+) and young children (<5). Tables S2 and S3 display the demographics of the online focus group participants, 43 of whom joined the 2020 sessions and 32 of whom joined the 2021 sessions. The 2020 sessions began with five sets with six to ten people in each group, and the 2021 sessions consisted of seven sets with three to seven participants in each group. About 81% and 56% of participants were White (non-Hispanic) and female, respectively.

### 3.2. Focus Group Themes

The three major themes that were derived to further explain the survey results were food safety perception, hand-cleaning, and cleaning food and thermometer use (Table 1). Codes categorized as ‘food safety perception’ expressed consumers’ food safety practices, awareness of foodborne illness, and opinions on food safety before and during the pandemic. ‘Hand-cleaning’ was expressed as consumers’ perceptions and practices of personal hygiene. These included handwashing practices, the use of sanitizer, and food delivery practices. The last theme, ‘cleaning food and thermometer use’ was related to consumers’ handling of food during the pandemic.

### 3.3. Food Safety Perceptions During COVID-19

**Figure 1:**
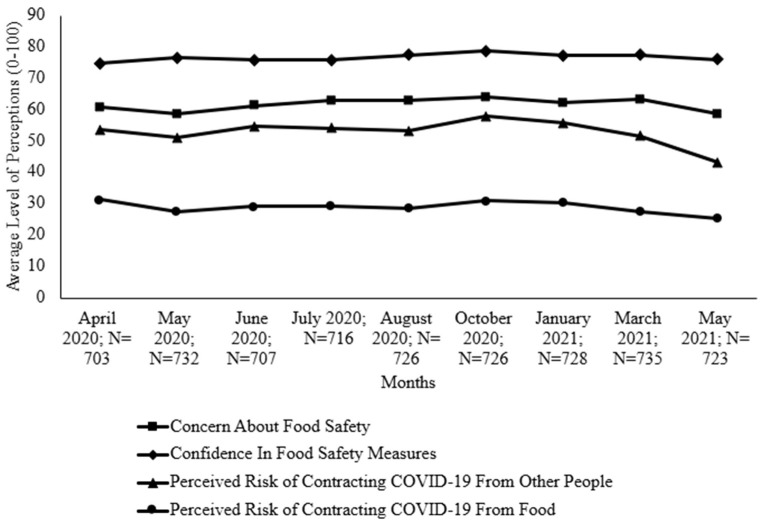
Food safety perceptions during COVID-19 (April 2020–May 2021). Significant results can be seen in Table S4.

Table S4 displays the mean scores (0–100) of the survey respondents’ food safety perceptions with significant differences throughout the pandemic, and Figure 1 displays a chart to visualize the trend. The concern about food safety fluctuated throughout data collection; however, the concern was lower in May 2020 than in October 2020 (Figure 1). Consumers’ confidence in their food safety measures ranged from 74.75 to 78.58 throughout the months but was significantly higher in October 2020 than in April 2020. For the months from April 2020 to March 2021, consumers were above “somewhat concerned” (50) about contracting COVID-19 from other people, but this average dropped to less than “somewhat concerned” in May 2021 (43.31), which was significantly lower than those in all other months. For all months, consumers had a low risk perception of contracting COVID-19 from food, with the lowest point in May 2021, significantly lower than those in April 2020, October 2020, and January 2020 (Table S3). When comparing the perceived risk of contracting COVID-19 from people in contrast to transmission from food, the perceived risk of contracting COVID-19 from people was significantly higher in all months (significance not shown in Table S4).

Earlier in the pandemic, some focus group participants were more concerned about contracting COVID-19 from other people than from food: “Not really food but … I am scared of getting it from other people, but not from food too much” (male, 35–44, May 2020). Similarly, many of the participants from the 2021 sessions were not as concerned about food safety in June 2021 as they were back when the pandemic had started: “In the beginning, like I said, I was very wary about touching anything, and then as time moved on, I said, ‘OK, I’ll eat the apple, [but] I’ll still wash it” (male, 55–64, June 2021). Those who were not concerned about contracting COVID-19 from food mentioned that it was so because the virus was airborne. Participants in both sessions (2020 and 2021) who were concerned about their food being contaminated with the virus were uneasy about the exposure of food to other people, exemplified by the following statement: “It is scary, though, when you go to the grocery store and see someone without a mask, breathing on peppers, picking it up, smelling it, and putting it back down again. Produce has still got to get washed” (female, 55–64, June 2021).

While participants in the 2020 sessions mentioned different practices to protect themselves from contracting COVID-19 from food, some focus group participants in the June 2021 sessions mentioned that everything was going “back to normal” regarding their food practices. This included how their grocery cleaning habits were more relaxed compared to those earlier in the pandemic: “When it first started … we would wash every single thing we bought no matter what it was. As time went on, it kind of became more ‘well, if this is going to sit in the fridge for a couple days or sit outside, if we are not going to touch this box of crackers for a few days,’ we didn’t worry about it” (female, 18–24, June 2021). Some participants from both the 2020 and 2021 sessions mentioned that they abided by food safety practices prior to the COVID-19 pandemic. However, one participant from the 2021 sessions claimed to implement these practices due to increased worrying about pesticides rather than from bacteria and viruses. Similarly, a few participants from the 2020 sessions indicated an intention to continue washing produce because of pesticides: “I do [wash fruits and vegetables] just for pesticide use. That was what I did before, and that’s what I continue to do it for” (female, 45–54, July 2020). One participant from the 2021 sessions also described how he also washed meat before cooking it (Table 1). While many participants said that they were aware of foodborne illness before the pandemic, a few noted that the pandemic had “heightened” their awareness (Table 1). While the concern of contracting COVID-19 from food was not high in either the surveys or focus groups, focus group participants still mentioned various techniques to reduce the risk of contracting COVID-19, foodborne illness, and ingesting pesticides from food. These techniques may or may not align with recommended food safety practices.

### 3.4. Handwashing

**Figure 2:**
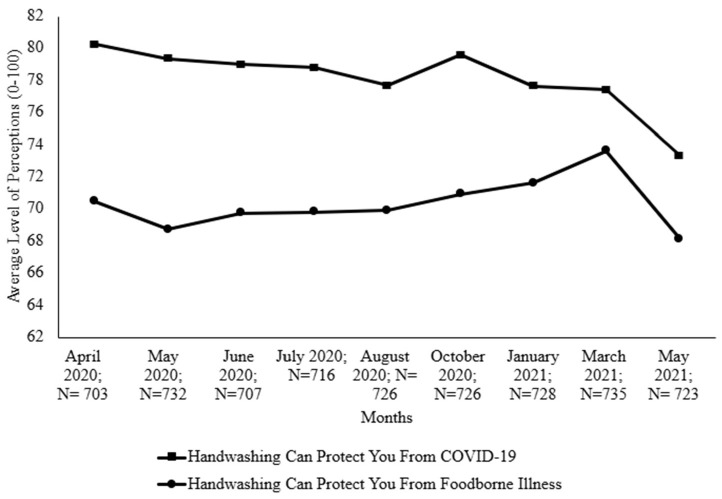
Belief in protective effect of handwashing (April 2020–May 2021). Significant results can be seen in Table S5.

Handwashing has been a key preventive measure against SARS-CoV-2 and, for years, it has been a key player in preventing foodborne illness [35]. Table S5 depicts average levels of handwashing perceptions and significant differences between each month, while Figure 2 is a chart through which we visualize the trend. During all points of data collection, consumers expressed a perception that handwashing was significantly less effective in protecting them from foodborne illness than it was in protecting against COVID-19. Consumers in May 2021 had significantly less confidence in the ability of handwashing to protect them from COVID-19 compared to all the other months except March 2021. Additionally, in May 2021, consumers had a significantly lower belief that handwashing protected them from foodborne illness than consumers in March 2021 did. The belief that handwashing offered protection from foodborne illness was higher in March 2021 than in May 2020 (Table S4).

**Figure 3:**
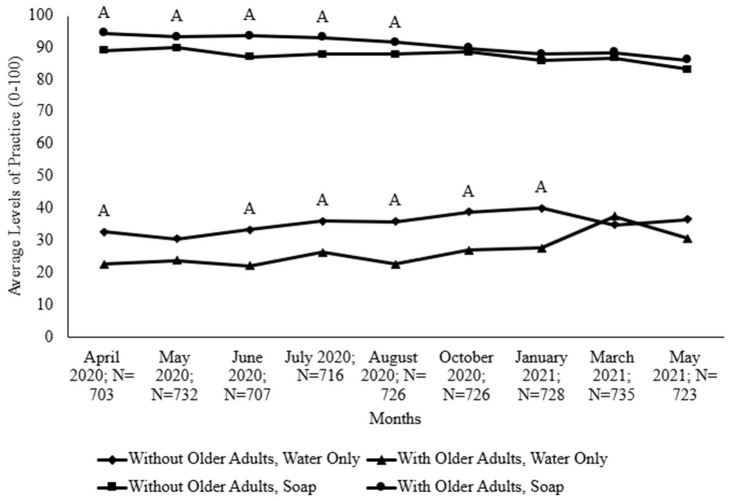
Comparing levels of handwashing between households that have or do not have older adults (age 65+). Uppercase letters indicate significant differences among handwashing practices (water only or soap) of those living with older adults and those living without older adults.

**Figure 4:**
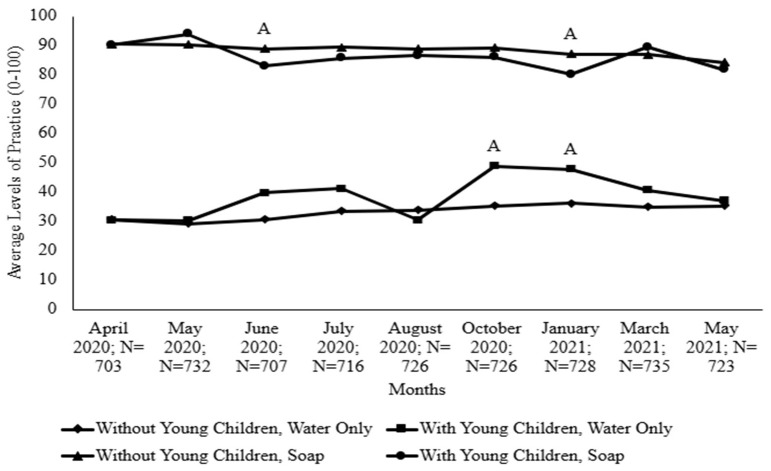
Comparing levels of handwashing between households that have or do not have young children (age < 5). Uppercase letters indicate significant differences between handwashing practices (water only or soap) among households with young children in comparison to those without young children.

To assess high-risk individuals, the researchers analyzed households with older adults (age 65+), as shown in Figure 3, and households that had young children, as Figure 4 illustrates. For all months except March 2021, households without older adults had higher levels of handwashing with water only, with significant differences in April 2020 and June 2020 to January 2021. Conversely, for all months, households with older adults had higher levels of handwashing with soap and water, with significant differences from April 2020 to August 2020.

Trends for both handwashing practices (water only and water with soap) were similar; households became increasingly similar in their levels of practice over time during the pandemic, as Figure 3 indicates. While both populations washed their hands more with soap and water when compared to water only in May 2020 and January 2021, Figure 4 shows that households without children had significantly higher levels of handwashing with soap than households with children did. In October 2020 and January 2021, households with young children practiced handwashing with water only at significantly higher levels than households without young children did.

While participants from both the 2020 and 2021 sessions mentioned handwashing, most reported performing so more to protect themselves from contracting COVID-19 rather than foodborne illness, exemplified by this participant’s comment excerpted from Table 1: “At home I constantly wash my hands. Even this morning, I was washing my hands and my wife called me, ‘you still wash your hands?’ I’m used to washing up. I’m already vaccinated. Yeah, but I’m still scared” (male 35–44, June 2021). One participant from the 2020 session even mentioned that prior to the pandemic, peer pressure in public restrooms caused her to wash her hands with soap, explaining the following: “So when I was at work, I have this peer pressure. You’re around people, and in a public restroom you must do the full hand wash … I was just pretty much a water-and-go person here in the house, and now I’m doing the whole happy birthday song with soap” (female, 45–54, May 2020). Focus group participants from the 2021 sessions mentioned that their practices were reverting to normal, but almost all participants from both sessions claimed that they would continue washing their hands after the pandemic. As Table 1 shows, they attributed this continuation to numerous reasons, including protection from other “germs”, maintaining a practice that they had established before the pandemic, and because it was generally a good practice to have. Many focus group participants indicated that they would stop using hand sanitizer because they never used it before, it was irritating to the skin, and/or because they considered the smell unpleasant.

### 3.5. Cleaning Food

**Figure 5:**
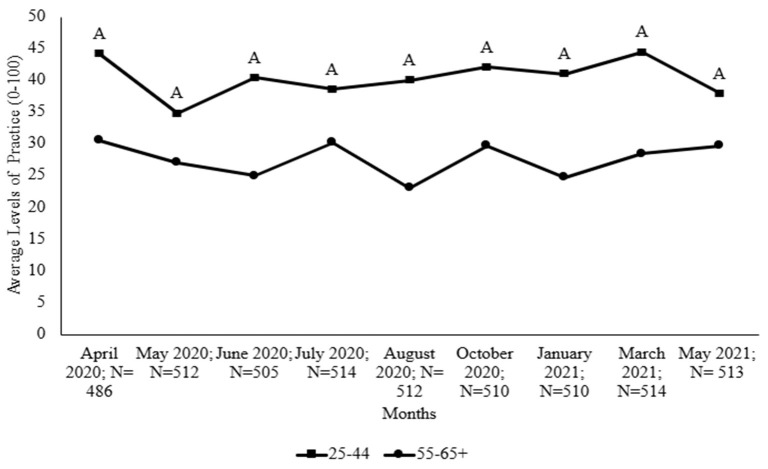
Levels of washing produce with soap among participants who were 25–44 years old and 55–65+ years old. The uppercase letter indicates a significant difference between age groups.

The findings revealed some differences in levels of produce-washing with soap among survey respondents in different age groups; as Figure S1 shows, distinctions were especially apparent between those who were 25–44 years old and those who were 55–65+ years old. Figure 5 shows that the younger age group (25–44 years) washed produce with soap at significantly higher levels than members of the older age group (55–65+).

While the focus group participants in 2021 said that they were resuming normal routines, participants from both sessions discussed the ongoing food safety practices that they used to prevent themselves from contracting COVID-19 from their food. Very few people acknowledged that they did not carry out any cleaning of food at all, and most participants from both the 2020 and 2021 sessions indicated that they would continue to wash their produce with water (Table 1). Although more people reported washing their produce with soap and other methods (vinegar and commercial fruit washes) during the 2020 sessions, one participant from the 2021 sessions acknowledged still washing fruits and vegetables with dish soap. When asked about their intention to continue washing their produce after the pandemic, participants in both sessions agreed that they would continue to perform so for various reasons, including to wash off residue from pesticides, to remove microorganisms, and as a good practice.

## 4. Discussion

### 4.1. Back-to-Normal Perceptions

The results from this study suggest that participants’ food handling practices were reverting to what they were pre-pandemic, and aspects of this change in behavior required assessment and explanation. Overall, the survey and focus group data suggested that the consumers were reverting from their risk perceptions and practices that they had associated with reducing the transmissibility of COVID-19 from food. When compared to the possibility of contracting the virus from other people, the data even highlighted that the consumers’ risk perceptions of contracting COVID-19 from food were also lower earlier in the pandemic. As the focus group participants mentioned, this initial low perception might have been because the SARS-CoV-2 virus is an airborne pathogen and not foodborne [36]. Previous research on the preventive practices of people in Spain during the 2009 influenza A (H1N1) pandemic found trends similar to those observed in the present study, a decrease in the adoption of preventive measures over the course of the pandemic [37].

Handwashing was a preventive control during the pandemic as well as in everyday food handling for the participants. During the entire duration of data collection, the consumers believed that handwashing protected them more from COVID-19 than from foodborne illness. However, the consumers’ confidence in the effectiveness of handwashing for both declined in the later months in comparison to the earlier months of data collection. According to a survey conducted on behalf of the American Cleaning Institute (ACI), Americans were still washing their hands frequently in 2021, but nine out of ten had made some type of change in their handwashing habits since the pandemic had begun [38]. Consumers may not have been aware of or concerned about contracting foodborne illness from unclean hands, which may have caused the decline in handwashing habits to continue later in the pandemic or after the end of pandemic. Similar results were reported by Olapeju and colleagues [39], whose investigation showed that handwashing practices had declined over time, especially among urban populations in sub-Saharan Africa. This observation was attributed to the likelihood of other preventive measures for COVID-19, such as the wearing of face masks being emphasized as a more effective strategy, and also the possibility of “pandemic fatigue” among the population. Pandemic fatigue has been described as “demotivation to follow recommended protective behaviors, emerging gradually over time and affected by a number of emotions, experiences, and perceptions” [40]. This phenomenon of pandemic fatigue was seen as the psychological state of most populations after multiple lockdowns and as the pandemic dragged on [41]. Pandemic fatigue was capable of wielding influence over behavioral changes as significant as other known variables including attitudes, knowledge, and behavior [42].

Along with perceptions and practices reverting to pre-pandemic levels, consumers’ confidence in their food safety measures was also significantly higher in October 2020 than in April 2020. One reason for this increase in confidence and decrease in risk perception might have been that consumers were letting their guard down after six months of the pandemic, evidenced by the spike in COVID-19 cases around October and November 2020 [43]. Along with the spike in cases, the number of people being vaccinated against COVID-19 around January 2021 was slowly increasing [44], which may explain the decreased risk perception. This finding is similar to that reported in Europe (in 16 countries), where individual risk perception decreased after vaccination, which was attributed to a change in risk perceptions as a result of access to preventive measures known as the Peltzman effect [45]. A study in Malaysia also reported similar findings [46], suggesting that vaccinations may have altered risk perceptions and as a result impacted initial preventive measures. Even though risk perceptions decreased in this current study as well, some focus group participants mentioned that the pandemic had heightened their awareness of foodborne illness. Even so, increased awareness and knowledge may not guarantee changes in practices or perceptions [47].

### 4.2. Caution Fatigue

Along with lower risk perceptions, levels of recommended food safety practices such as washing hands with soap and washing produce with water decreased over time. This may be explained by lower risk perceptions of contracting COVID-19 from food and by “caution fatigue” which, according to experts, is like an aging AA battery; people feel energized and ready to combat the virus earlier in the pandemic, but as time goes on, they feel depleted and have low motivation to stay safe [48,49]. The lower levels of practices also might be due to individuals’ optimism bias or the belief that negative consequences are less likely to occur to oneself when compared to others [50]. This bias can cause consumers to engage in unsafe practices and ignore public health warnings [51]. Similar findings were reported in India among emerging adults where caution fatigue was one of the factors predicted to affect the course of the response to the pandemic over time [52]. Ju and Downey [53] confirmed the presence of optimism bias among US consumers during the pandemic. A study in Thailand also reported on optimism bias impacting adapted preventive measures among older adults during the pandemic [54]. It was highlighted that optimism bias was further associated with socioeconomic and poverty- and health-related factors.

### 4.3. Reaching Different Demographics

Demographics have been widely reported as a significant predictor of food safety behaviors during COVID-19 [55,56,57]. The data from the present study indicate that differences in demographics, especially age groups, could cause people to adopt or change certain hand hygiene and food handling behaviors. Washing produce with soap was significantly higher among the younger age groups (25–44 years) than the older age groups (55–65+). Haas and colleagues [58] reported a similar trend among study participants, voicing a concern about the practice of washing produce with soap or other substances during the pandemic. The difference between age groups may be due to the increased use of social media among the younger generation of consumers [59]. A previous content analysis study of social media platforms, such as YouTube, during the beginning of the pandemic found that some of the content consisted of misinformation, advocating poor food handling procedures, such as washing produce with soap [60]. Previous studies showed that consumers adopt practices from what they see or read [15,61]. Because information can be spread quickly digitally, especially among younger consumers, “ill-advised” practices like washing produce with soap may have been adopted before science-based information was widely distributed.

Another demographic factor that was highlighted in this study was the households’ composition. The food hygiene practices in households with high-risk individuals were significantly different from those in those without. The Centers for Disease Control and Prevention [62], as well as the U.S. Food and Drug Administration [63], indicate older adults (65+) and younger children (ages < 5) as at-risk populations for foodborne illnesses, as well as other diseases including COVID-19. Food handling practices that are not well managed in households with such high-risk populations may pose a health threat to these groups. Policymakers not only need to be attentive to details concerning individuals when developing risk communication initiatives, but they also must investigate the possible impact of household compositions.

### 4.4. Consumer Behavioral Change over Time

This study revealed significant changes in consumer behavior, particularly in the adoption of certain food handling practices during the pandemic’s onset and their subsequent reversion to pre-pandemic behaviors as time progressed. The Health Belief Model (HBM), a widely used theoretical framework in consumer behavioral studies, provided valuable insights into understanding these behavioral patterns during the pandemic [64,65,66,67]. A study by Alagarsamy and colleagues [68] reported perceived threats and cues to action as HBM constructs which impacted Indian consumers’ behavioral intent to buy organic foods during the pandemic, leading to changes in dietary patterns. The concept of perceived severity within the HBM is particularly relevant to our findings among US consumers. This construct, which represents how seriously individuals view the consequences of not changing their behavior, helps explain the observed behavioral shifts during and after the pandemic. During the height of the pandemic, the perceived severity of COVID-19 likely motivated enhanced food handling practices. However, as the perceived threat diminished over time, consumers appeared to revert to their pre-pandemic practices.

Cues to action, another key HBM construct, manifested through social influence and media coverage during the pandemic. Alagarsamy and colleagues [68] documented how these cues stimulated behavioral changes among Indian consumers, particularly in their intentions to purchase organic foods. In Ghana, similar findings were also reported on the impact of media information on behaviors during the pandemic [69]. However, the abundance of misinformation in public discourse led to some uninformed decisions. In the current study, this phenomenon was particularly evident in practices such as washing produce with soap, which became prevalent among certain age groups, potentially due to the proliferation of non-scientific content on the internet.

Another HBM construct that could explain consumers’ behavior is self-efficacy. Consumer confidence was increasing and risk perception decreasing six months into the pandemic, which could have stemmed from confidence in themselves in performing preventive measures and also as result of vaccinations which peaked around that time. Supporting this interpretation, research in Iran demonstrated that self-efficacy was a key predictor of preventive health behavior for COVID-19 among adolescents [70]. This finding suggested that an effective measure was to boost health literacy by providing more knowledge to these adolescents to increase their self-confidence in adopting health behaviors.

### 4.5. Practical Recommendations and Future Directions

This study’s findings regarding food handling practices across demographic groups suggest several important policy and educational implications.

- The heterogeneity in food safety behaviors across different demographic segments necessitates the development of targeted intervention strategies. We propose implementing demographically tailored communication campaigns that account for the specific needs, preferences, and behavioral patterns of different population subgroups. The effectiveness of such targeted approaches requires careful consideration of communication channels and platforms that are most accessible and appealing to each demographic segment. In our increasingly digital society, various social media platforms demonstrate distinct demographic affinities, suggesting the need for strategic platform selection in educational outreach efforts.
- To enhance the sustainability and reach of food safety initiatives, we recommend integrating these campaigns into existing public health policy frameworks. Current public health policies encompass mandatory components in school curricula, worker training programs, and community initiatives [71]. By incorporating evidence-based food safety education into these established frameworks, policymakers can ensure broader exposure to scientifically sound information, thereby facilitating more informed decision-making regarding food handling practices. This integration approach can leverage existing institutional structures while promoting consistent and comprehensive food safety messaging.
- Furthermore, the significant role of social media in contemporary society presents both challenges and opportunities for public health communication. We recommend that public health educators and stakeholders develop comprehensive digital communication strategies that leverage these platforms effectively to disseminate timely, science-based information during public health events such as pandemics.

These recommendations emphasize the importance of evidence-based, strategically targeted interventions that can be effectively integrated into existing policy frameworks while adapting to contemporary communication channels. Success in implementing these recommendations will require ongoing collaboration between researchers, policymakers, educators, and public health practitioners to ensure that food safety messages reach and influence their intended audiences effectively.

Future research directions should explore strategies to sustain certain consumer food safety behaviors over time, particularly focusing on longitudinal studies that assess the integration of technology in food safety education initiatives. Several technological approaches such as web-based platforms and simulations, interactive modules and social media platforms can be leveraged to disseminate food safety information and promote essential practices such as proper handwashing with water and soap, voiding the use of soap when washing produce, and preventing cross-contamination between raw and ready-to-eat foods. The effectiveness of these technological interventions in promoting long-term behavior change should be investigated through extended studies. The data collected from such longitudinal studies will provide valuable insights to policymakers and educators in developing effective communication tools for food safety as well as public health initiatives.

## 5. Conclusions

This yearlong study yielded valuable findings about the impact that a global health event could have on food handling practices and food safety perceptions in the context of the COVID-19 pandemic. While consumers’ risk perceptions and food handling practices were heightened at the beginning of the pandemic, their initial diligence dwindled as the pandemic progressed. The causes of the “back-to-normal” phenomenon can be very complex, since human behavior is not just an outcome of knowledge or awareness. We observed that demographics such as age group and household compositions influenced food safety practices adopted during the pandemic. The food safety handling practices of young adults were different from those of elderly people. Households with high-risk individuals also were found to have significant differences in practices compared to those without. The findings highlighted the need for health communicators, researchers, and food safety educators to investigate various factors when developing effective risk communications for food safety handling practices during events such as pandemics. Consumers’ food handling practices change over time, and initiatives may need to evolve accordingly to help ensure the continual adoption of these practices. Responsible agencies and stakeholders also need to reach different demographic groups through different platforms with science-based information to ensure that the right information is shared and, consequently, proper food safety practices are adopted.

## 6. Limitations

Although this study was carefully designed and executed by the researchers, limitations do exist. The participants may not have represented all U.S. consumers due to the restrictive conditions of the COVID-19 pandemic and the online nature of the recruitment process. Even though some volunteers did not participate in the survey at every point in time, all participants were recruited from the same pool with defined inclusion criteria which ensured that a representative group was sampled each time. Despite variations in the populations, the large sample size helped to gain insights about observed changes in consumer practices. Consumers with limited or no internet access may not have been represented. Future research can benefit from using multiple recruitment strategies, including phone calls or in-person methods.

Due to limited resources, the focus group sessions could not be conducted for every single month. However, the researchers did determine that differences among responses from the focus group sessions in the first three consecutive months were insignificant. Therefore, resources were prioritized in the extended survey instead of pairing it with focus groups. In addition, those who participated were volunteers and may have had an interest in the topic, so their practices and perceptions may differ from those who have less interest. Also, some focus group participants dropped out or did not show up to the sessions. Failure to attend may have been attributable to problems with internet connectivity, differing time zones, scheduling conflicts, or even loss of interest. Lastly, because these data was self-reported, some discrepancies may have occurred between what consumers say and what consumers actually carry out regarding their practices. Further observational or similar methods can be explored in future consumer studies.

## Figures and Tables

**Table 1:** Focus group codes and themes and representative quotes from participants.

Code	Example Quote(s)	Theme
Not concerned about food	“Not really food but … I am scared of getting it from other people but not from food too much” (male, 35–44, May 2020). “I thought in the beginning, like I said, I was very wary about touching anything, and then as time moved on, I said okay, I’ll eat the apple, [but] I’ll still wash it” (male, 55–64, June 2021).	Food Safety Perception
Exposure to food	“Because the times I been in the store people have been walking around touching everything and moving on, just leaving it there and, you know, I’m just not comfortable with that” (female, 65+, June 2020) “It is scary though when you go to the grocery store and see someone without a mask, breathing on peppers, picking it up, smelling it, and putting it back down again. Produce has still got to get washed” (female, 55–64, June 2021).	
Back to normal *	“I think just because of my lack of knowledge, I was definitely more careful. I wiped down my groceries when I got home from the store, I would leave them in the garage for two or three days to, like, disinfect or whatever. Honestly, that didn’t last long, that lasted about three weeks and I’m like, this is utterly ridiculous. So, then I honestly just pretty much went back to my normal food habits. So, nothing, no extra precautions because of COVID” (female, 45–54, June 2021). “I think everything is normal here. People wear masks sometimes, people don’t sometimes, but everything is pretty much open, and I don’t feel very affected at all” (male, 45–54, June 2021).	
Cleaning groceries	“Yeah, I do actually use the dish soap and I have an antibacterial dish soap and I just wash it off and I take a paper towel and dry it. Like if it’s an apple, for example, but if it’s a banana I don’t do that. Anything that I know that somebody else has touched the surface I would wash all the produce. For example, I have not washed the outside of an avocado because when they cut it open…there’s just certain things that…celery lettuce those are the things that I would wash” (female, 45–54, June 2020) “When it first started, I remember I was living with my parents, [and] we would wash every single thing we bought no matter what it was. As time went on, it kind of became more ‘well, if this is going to sit in the fridge for a couple days or sit outside, if we are not going to touch this box of crackers for a few days,’ we didn’t worry about it” (female, 18–24, June 2021).	
Heightening awareness of foodborne illness *	“Certainly, COVID made it heightened. Absolutely, for me it heightened my awareness. Before, I probably [would] eat anything if I went into a supermarket. I might even grab a grape and steal a grape. Now I won’t do that anymore. I’m very, very cautious” (male, 65+, June 2021). “I think for us it did make us more aware because neither of us have backgrounds in food or anything like that, so it did make us aware” (male, 65+, June 2021).	
Awareness of foodborne illness	“I would say that I pretty much been aware of, you know, foodborne illness most of my life. I mean I grew up on a ranch and so you know. You talked about, you know, you got to cook your pork, you know, this and that” (male, 55–64, July 2020) “Yes, I’ve always been especially conscious about foodborne illness. My mother was a dietitian and former food inspector, so I grew up with making sure I temp [check the temperature of] everything and that everything is cooked, and everything is handled appropriately, so it’s just second nature to me” (female, 35–44). “I’m completely guilty of not washing stuff in between because I just don’t think about it, but all the same, you see just as many reports about E. coli, spinach and stuff like that. I probably should, like, wash my vegetables longer and not just do the, run it under the sink real quick. But outside of that, I mean I check expiration dates because I did notice that especially during COVID, like, I don’t know if they were just short employees at the store or what, but there was a few times when I got stuff home and it was already expired. They just weren’t pulling stuff off the shelves” (female, 35–44).	
Food safety practices pre-COVID-19	“Yeah, I think we wash fruits and vegetables—it’s just something that we’ve always done and seems like the right thing to do before COVID as well” (female, 65+, July 2020) “I just washed it with regular dish soap. I know it’s probably not that effective but it’s what I’ve always done” (male, 55–64). “I think with produce, I’m actually more concerned about pesticides and things like that than I am contamination of viruses or *E. coli* or whatever. I try to buy organic whenever I can. And when I buy organic, I just rinse it off under the sink. Anything that’s not organic, and I don’t know how effective it is. I’ll buy that fruit and veggie wash that’s supposed to remove most of the pesticides from it. So, I would definitely say my concerns about pesticides and things like that are higher than my concerns about catching some foodborne illness from produce” (female, 45–54).	
Hand-cleaning beyond COVID-19	“I think it’s kind of like habit forming. I don’t think it’s a bad thing to wash your hands a lot so [I’ll] probably keep doing it” (female, 25–34, June 2020) “Good hand washing is personally the way to go. I mean, yeah, I’ve been using hand sanitizer more frequently, but it cannot replace hand washing, you know, I don’t think it ever will” (male, 35–44, June 2021).	Hand-Cleaning
Practices	“I’m still very diligent in washing my hands and using the hand sanitizer” (female, 45–54, July 2020). “At home I constantly wash my hands. Even this morning, I was washing my hands and my wife called me ‘you still wash your hands’ I’m used to washing up. I’m already vaccinated. Yeah, but I’m still scared” (male, 35–44, June 2021).	
Hand sanitizer woes	“The sanitizer breaks my hands out, so I try to wash my hands more often. The alcohol really breaks my hands out” (female, 65+, May 2020). “I did not use hand sanitizer before. I really never used before, during, and I won’t use after. I’m very conscious about what I put on my skin because of what you put on your skin, you absorb into your body. I don’t freak out about it, but I would prefer that I don’t use a lot of chemicals on my body” (female, 35–44, June 2021). “I agree with [other participant on] the hand sanitizer. I don’t really like it. I never liked it. It, it dries my skin out and I really hate the smell. So I probably definitely won’t do that” (male, 35–44, June 2021).	
Food delivery and takeout practices	“[I] use the hand sanitizer and try to … the two times that I got takeout, they didn’t set it down, they handed it to me, but I avoided touching their hands and then used hand sanitizer right away” (male, 55–64, May 2020). “When I order takeout, I would leave the outer bag outside and take the inner bag inside, and sometimes I would transfer food to another container and wash my hands thoroughly” (female, 55–64, June 2021).	
Cleaning groceries	“I’ve been washing, you know, produce and vegetables and fruit. I mean it’s just like running water and a kitchen brush. I don’t use soap or anything. Just run it through water for a minute or so” (male, 45–54, June 2020). “I used to just use water sometimes and then if I’m on the go sometimes, you know, I forget to wash my fruit, but with COVID I’ve been more careful and been washing my produce with just water. But before then I wasn’t really doing that as much” (female, 25–34, June 2021). “I just washed it [fruits and vegetables] with regular dish soap. I know it’s probably not that effective but it’s what I’ve always done (male 55–64, June 2021)	Cleaning Food and Thermometer Use
Not cleaning groceries	“I don’t sanitize my food much and I still get fresh fruits and vegetables. My whole thing is if, you know, I sanitize my hands after getting the things before and after, I feel relatively safe as they say this is more of an airborne disease than spread by hands, even though it can possibility get on the stuff by airborne” (male, 45–54, July 2020) “Early on I was reading things about people need to wash down the boxes that the cereal came in and bleach your food and vegetables and that sort of thing. I never did any of that” (male, 65+, June 2021). “No, I did that. I did that [wiping down packages] for the first two weeks, and then I said, this is ridiculous” (male 55–64, June 2021).	
Cleaning produce beyond COVID-19	“Yeah, it’s all going to become second nature. It’s just going to be in our routine now” (male, 45–54, July 2020). “I definitely feel like I needed to be more cognizant of it because I do to worry about pesticides, and then there’s also the risk of E. coli and stuff like that because places don’t do proper handling of food. So, I don’t know that I would buy the fruit and vegetable wash. I’ve tried it before and I wasn’t sure if it made a difference or not, but I definitely know that just sticking, like, a zucchini under the water for, like, five seconds, isn’t cutting it” (female, 35–44, June 2021). “Yeah, I think I’ll keep using the soap for a while at least just to be safe” (male, 18–24, June 2021).	
Cooking to kill the virus	“If it can be cooked, it’s cooked. I’m assuming that temperature will help kill virus so we’re cooking everything that can be cooked” (female, 55–64, May 2020). “I may have already mentioned it, but I would get food that I could reheat in the microwave because I figured that the microwave would kill any viruses before the food got too hot, so my main safety precaution with restaurant food was just reheating” (female, 18–24).	

* This code was not used in the 2020 sessions.

## Data Availability

The original contributions presented in the study are included in the article, further inquiries can be directed to the corresponding author.

## Supplementary Materials

The following supporting information can be downloaded at https://www.mdpi.com/article/10.3390/foods14040551/s1, Figure S1: Levels of washing produce with soap for different age groups (April 2020–May 2021); Figure S2: Levels of hand hygiene during COVID-19 (April 2020 to May 2021); Figure S3: Levels of produce washing during the COVID-19 pandemic (April 2020–May 2021); Table S1: Demographic information of respondents from 9 waves of surveys (April 2020 to May 2021); Table S2: All focus group members’ demographics in 2020 sessions; Table S3: Focus group demographics in 2021 sessions (June 2021); Table S4: Food safety perceptions during COVID-19 (April 2020–May 2021); Table S5: Belief in protective effect of handwashing (April 2020–May 2021); Table S6: Levels of hand hygiene during COVID-19.; Table S7: Levels of produce washing during the COVID-19 pandemic (April 2020–May 2021).
