# Supplementary material for: The Yearlong Effect of COVID-19 on Food Safety: Consumer Practices and Perceptions Using Longitudinal Consumer Surveys and Focus Groups
Source: Foods. 2025 Feb 7;14(4):551. doi: 10.3390/foods14040551 (PMC11854136; doi:10.3390/foods14040551)
Supplement: Supplementary file 1 [file foods-14-00551-s001.zip › foods-3396664-supplementary.pdf]

The Year-Long Effect of COVID-19 on Food Safety: Consumer Practices and Perceptions Using Longitudinal Consumer Surveys and Focus Groups:
Supplemental Tables and Figures

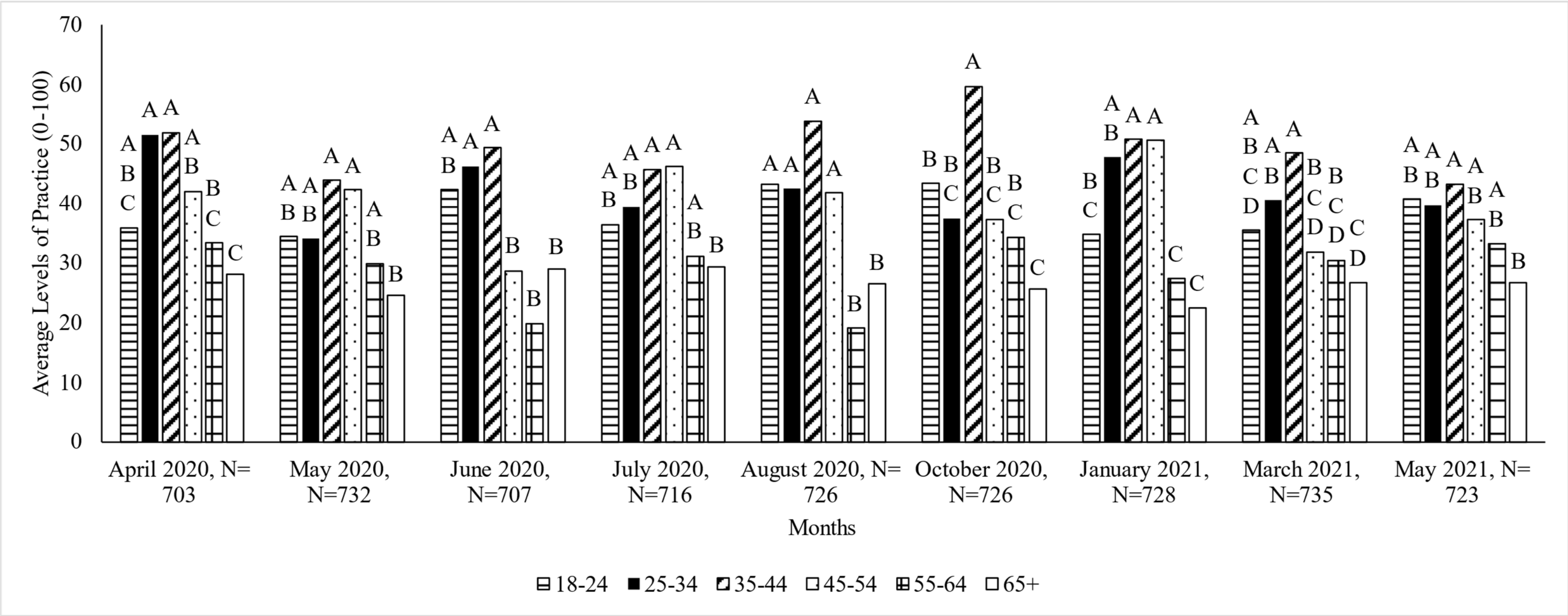

Figure S1. Levels of washing produce with soap for different age groups (April 2020-May 2021).

The bars in the graph represent the individuals within the 6 age groups in the present study (in numerical order): white bars with horizontal lines are 18-24 years old, solid black bars are 25-34 years old, white bars with slanted black lines are 34-44 years old, white bars with black dots are 45-54 years old, white bars with horizontal black lines + one vertical black line are 55-64 years old, and solid white bars are 65+ years old. The differences in stacked capital letters indicate significant differences between age groups within each month.

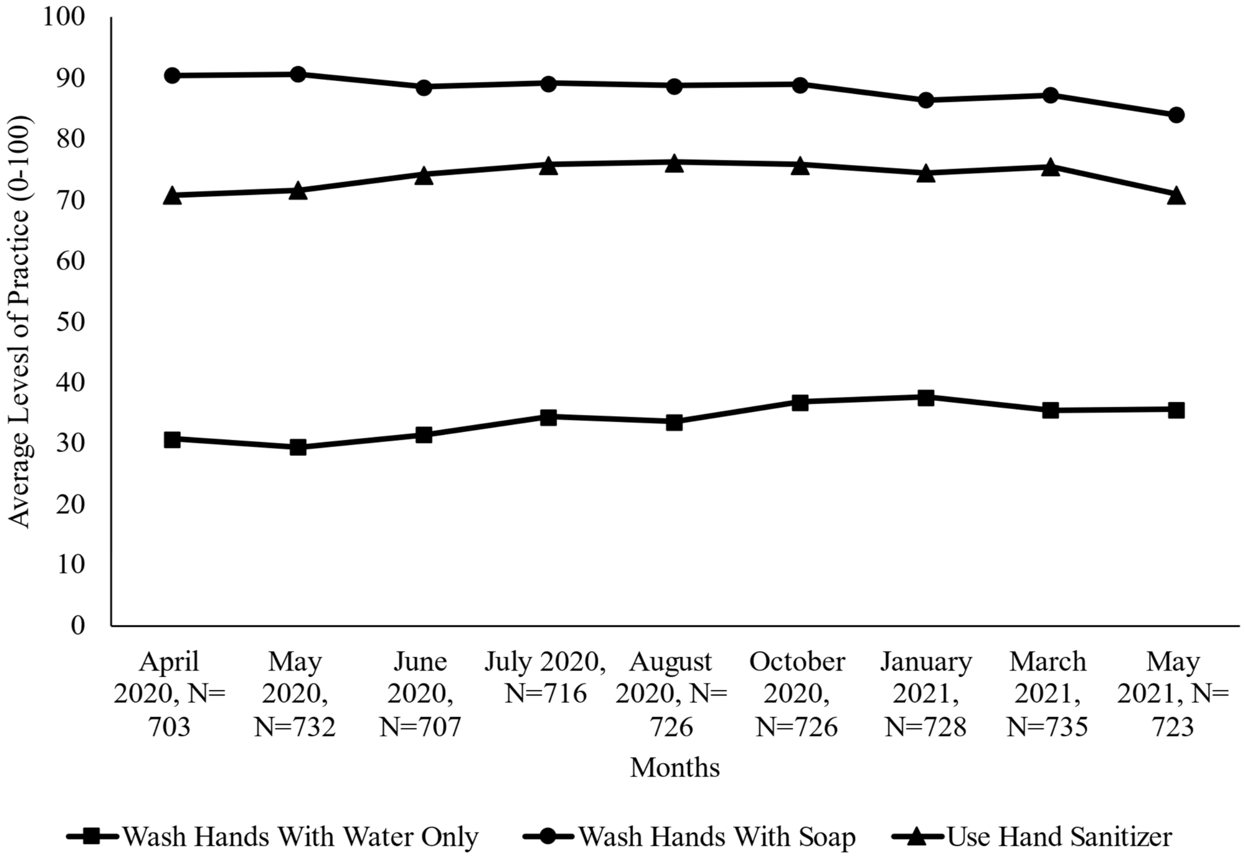

Figure S2. Levels of hand hygiene during COVID-19 (April 2020 to May 2021). Significant results can be seen in Table S6.

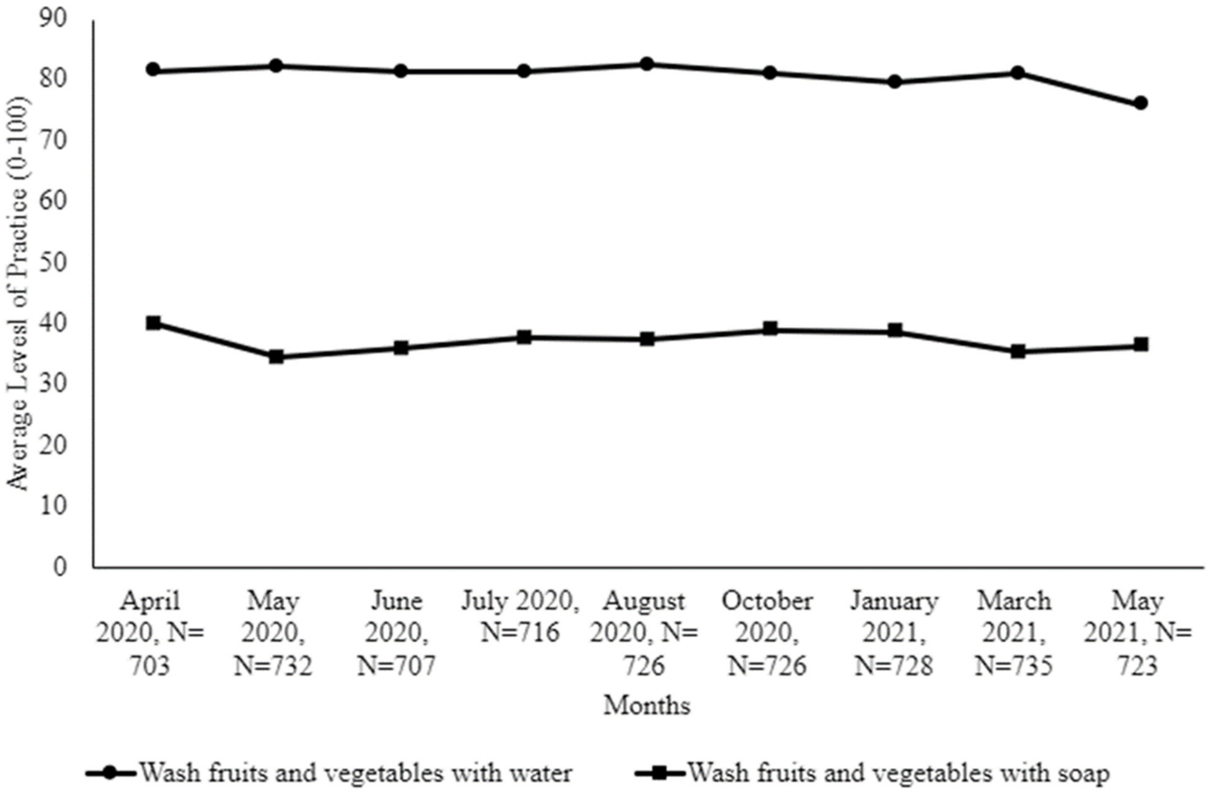

Figure S3. Levels of produce washing during the COVID-19 pandemic (April 2020–May 2021). Significant results can be seen in Table S7.

Table S1. Demographic information of respondents from 9 waves of surveys (April 2020 to May 2021)

	April 2020, N=703	May 2020, N=732	June 2020, N=707	July 2020, N=716	August 2020, N=726	October 2020, N = 726	January 2021, N=728	March 2021, N=735	May 2021 N= 723
Characteristics	%(n)	%(n)	%(n)	%(n)	%(n)	%(n)	%(n)	%(n)	%(n)
Gender									
Female	51(358)	51(375)	53(375)	52(375)	50(365)	51(372)	51(369)	51(375)	52(374)
Male	49(345)	49(357)	47(332)	48(341)	50(361)	49(354)	49(359)	49(360)	48(349)
Age									
18-24	12(86)	13(94)	13(95)	13(95)	13(92)	13(91)	13(95)	12(91)	13(95)
25-34	18(127)	18(131)	19(131)	18(131)	18(129)	18(129)	18(130)	18(131)	18(130)
35-44	15(102)	17(123)	17(123)	17(123)	17(123)	17(122)	16(120)	17(123)	17(123)
45-54	19(131)	17(126)	15(107)	15(107)	17(122)	17(125)	17(123)	18(130)	16(115)
55-64	17(119)	16(119)	16(112)	17(121)	17(121)	17(120)	17(121)	17(121)	17(121)
65 and above	20(138)	19(139)	20(139)	19(139)	19(139)	19(139)	19(139)	19(139)	19(139)
Ethnic									
White(non-Hispanic)	81(571)	76(556)	76(539)	81(581)	78(566)	82(593)	82(594)	79(577)	73(525)
Hispanic	6(41)	10(73)	6(39)	5(38)	7(53)	4(31)	5(35)	6(47)	9(62)
Black or African American	8(53)	8(60)	9(65)	8(55)	8(59)	7(53)	8(55)	8(55)	10(72)
Asian or Pacific Islander	5(32)	5(35)	7(46)	3(25)	4(29)	4(29)	3(25)	35(37)	6(44)
Other	1(4)	1(7)	2(15)	1(10)	2(11)	2(13)	2(13)	2(12)	2(16)
Native American	0(2)	0(1)	0(3)	1(7)	1(8)	1(7)	1(6)	1(7)	1(4)
Income									
Less than $10,000	6(42)	6(47)	6(43)	5(36)	8(56)	6(43)	7(48)	7(49)	8(56)

$10,000-$29,999	22(156)	19(138)	21(146)	22(156)	21(153)	20(145)	19(135)	22(163)	24(175)
$30,000-$49,999	19(130)	19(141)	19(133)	19(139)	18(129)	16(119)	18(134)	21(152)	20(143)
$50,000-$79,999	23(159)	24(176)	24(168)	24(169)	23(165)	21(149)	20(144)	22(162)	26(187)
$80,000 and above	28(197)	28(208)	27(192)	28(199)	27(195)	34(250)	33(237)	25(183)	21(150)
Prefer not to answer	3(19)	3(22)	4(25)	2(17)	4(28)	3(20)	4(30)	4(26)	12(2)
Education									
Not High School Graduate	2(14)	2(13)	1(10)	3(24)	3(22)	2(13)	1(9)	5(26)	4(32)
High School or GED Degree	38(269)	41(298)	40(281)	41(290)	44(316)	35(255)	38(277)	45(327)	48(350)
Bachelor's Degree	39(277)	39(282)	38(266)	33(238)	32(233)	35(253)	34(249)	31(229)	32(230)
Graduate Degree	19(132)	17(123)	18(130)	21(149)	19(139)	26(190)	25(180)	19(136)	13(92)
Prefer not to answer	2(11)	2(16)	3(20)	2(15)	2(16)	2(15)	2(13)	2(17)	3(19)
Experience in preparing meals									
Less than 1 year	1(8)	2(13)	1(9)	2(13)	2(16)	2(14)	2(15)	3(20)	3(21)
1-3 years	8(57)	7(50)	9(61)	9(68)	8(59)	10(74)	12(85)	11(82)	9(66)
3-5 years	9(60)	8(56)	8(60)	7(53)	8(55)	10(71)	9(69)	9(65)	9(63)
Over 5 years	82(578)	84(613)	82(577)	81(582)	82(596)	78(567)	77(559)	77(568)	79(573)
Total people in the household									
1	28(198)	30(216)	27(193)	26(186)	24(175)	24(177)	24(178)	26(193)	24(170)
2	33(229)	30(221)	33(232)	33(239)	32(232)	28(203)	30(222)	30(217)	35(256)
3	18(123)	18(128)	18(124)	16(111)	18(130)	16(113)	15(110)	17(127)	19(135)
4	15(105)	15(107)	15(109)	15(104)	15(110)	22(158)	20(143)	15(113)	12(90)
5	5(32)	5(37)	4(31)	8(56)	8(57)	8(56)	6(45)	8(56)	7(48)
More than 5	2(16)	3(23)	3(18)	3(20)	3(22)	3(19)	4(30)	4(29)	3(24)

Conditions of people living in the household									
Children younger than age 5	5(36)	9(66)	8(58)	10(69)	11(79)	11(77)	11(79)	10(72)	11(77)
People age 65 and over	21(145)	19(138)	18(130)	19(136)	18(134)	18(130)	21(151)	19(142)	21(151)
Diabetes	15(105)	13(93)	14(102)	14(102)	14(99)	16(114)	15(110)	16(114)	15(106)
Lung conditions	7(48)	6(43)	7(48)	8(59)	6(45)	7(53)	7(48)	8(57)	8(60)
Liver or kidney diseases	3(18)	2(12)	3(18)	3(23)	2(14)	2(16)	3(24)	3(25)	3(20)
HIV/AID	0(2)	0(2)	1(4)	0(2)	1(6)	0(1)	1(7)	1(7)	1(10)
Cancer	3(22)	3(19)	3(23)	2(17)	3(25)	3(20)	2(14)	4(29)	4(30)
Immunocompromised, including organ transplant patients	3(22)	2(18)	4(26)	4(27)	1(10)	2(18)	2(16)	3(20)	3(22)
At least 1 person in the household is at -risk	37(263)	38 (279)	38 (268)	40 (287)	38 (276)	42 (307)	41 (322)	42 (304)	45 (322)

Table S2. All focus group member demographic in 2020 sessions

		LM (n=10)	H1 (n=8)	H2 (n=10)	H3 (n=9)	H4 (n=6)	Total
Age							
	18-24	2	0	3	1	0	6
	25-34	2	0	0	5	0	7
	35-44	2	1	0	0	0	3
	45-54	1	4	1	2	3	11
	55-64	2	1	3	1	1	8
	65+	1	2	3	0	2	8
Ethnicity							
	White/ non-Hispanic	9	7	8	8	3	35
	Black or African American	0	1	1	0	2	4
	Hispanic	1	0	1	1	0	3
	Asian or Pacific Islander	0	0	0	0	1	1
Gender							
	Male	5	4	2	5	3	19
	Female	5	4	8	4	3	24

LM Represents the focus group with participants from low-medium states for COVID-19 cases on April 27, 2020 (under 10,000).

H Represents the focus groups (1–4) with participants from the high states for COVID-19 cases on April 27, 2020 (over 10,000 cases). Some participants

Table S3. Focus group demographic in 2021 Sessions (June 2021)

		S1 (n=4)	S2 (n=5)	S3 (n=4)	S4 (n=3)	S5 (n=5)	S6 (n=7)	S7 (n=4)	Total (n=32)
Age									
	18–24	2	0	0	1	0	0	0	3
	25–34	0	1	0	0	1	0	1	3
	35–44	0	1	3	0	0	2	1	7
	45–54	0	0	1	0	0	1	0	2
	55–64	1	2	0	2	4	1	2	12
	65+	1	1	0	0	0	3	0	5
Ethnicity									
	White (non-Hispanic)	2	5	5	3	4	6	3	28
	Black or African American	1	0	0	0	0	0	0	1
	Hispanic	1	0	0	0	1	1	1	4
	Asian or Pacific Islander	0	0	0	0	0	0	0	0
Gender									
	Male	2	2	2	0	1	7	1	15
	Female	2	3	2	3	4	0	3	17

S1 through S7 indicate the 7 different sessions that occurred in June 2021

Table S4. Food safety perceptions during COVID-19 (April 2020-May 2021)
[0=Not concerned at all, 50=Somewhat concerned,100=Very concerned]

	**April 2020 N= 703**	**May 2020 N=732**	**June 2020 N=707**	**July 2020 N=716**	**August 2020 N= 726**	**October 2020 N=726**	**January 2021 N=728**	**March 2021 N=735**	**May 2021 N= 723**
Concern about food safety	60.71 ± 29.73 [ab]	58.71± 30.98[b]	61.35 ± 30.48 [ab]	63.02 ±29.38 [ab]	62.79 ± 30.54 [ab]	63.89 ± 30.66 [a]	62.10 ±30.52 [ab]	63.41± 30.68 [ab]	58.74 ± 32.73 [ab]
Confidence in food safety measures	74.75 ±21.34 [b]	76.43± 21.21[ab]	75.97 ± 21.66 [ab]	75.87± 21.23 [ab]	77.47 ±21.35 [ab]	78.58 ± 21.83 [a]	77.28 ± 21.08 [ab]	77.42 ±21.99 [ab]	76.02 ±23.43 [ab]
Perceived risk of getting COVID-19 from other people	53.68 ± 28.17 [ac]	51.11 ± 28.78[c]	54.68 ± 29.87 [ac]	54.15 ± 28.88[ac]	53.21 ± 29.31[ac]	57.79 ± 29.53 [a]	55.79 ± 28.85 [a]	51.67 ±29.78 [b]	43.31 ± 30.77[d]
Perceived risk of getting COVID-19 from food	31.38 ± 27.27 [a]	27.56 ± 25.97 [ab]	29.14 ± 26.79 [ab]	29.47 ± 27.35 [ab]	28.75 ± 27.5 [ab]	30.98 ± 29.74[a]	30.51 ± 29.43 [a]	27.54 ±27.88 [ab]	25.39 ±27.42 [b]

Different superscripts indicate significant differences between months

Table S5. Belief on protective effect of handwashing (April 2020- May 2021)
[0= Do not believe, 50= Believe moderately, 100= Believe completely]

	**April 2020 N= 703**	**May 2020 N=732**	**June 2020 N=707**	**July 2020 N=716**	**August 2020 N= 726**	**October 2020 N=726**	**January 2021 N=728**	**March 2021 N=735**	**May 2021 N= 723**
Handwashing can protect you from COVID-19	80.23 ± 20.50^{a}	79.36 ± 22.25^{a}	79.00 ± 21.43	78.79 ± 23.02^{a}	77.68 ± 23.64^{a}	79.59 ± 22.43^{a}	77.64 ± 23.31^{a}	77.43 ± 23.98^{ab}	73.34 ± 26.84^{b}
Handwashing can protect you from foodborne illness	70.50 ± 27.12^{abc}	68.75 ± 29.05^{b}	69.76 ± 27.48^{abc}	69.84 ± 29.02^{abc}	69.92 ± 28.31^{abc}	70.95 ± 28.29^{abc}	71.63 ± 27.60^{abc}	73.65 ± 26.24^{a}	68.16 ± 29.61^{bc}

Different superscripts indicate significant differences between months

Table S6. Levels of hand hygiene during COVID-19
[0= Never, 50= Sometimes, 100 = Always]

	April 2020 N= 703	May 2020 N=732	June 2020 N=707	July 2020 N=716	August 2020 N= 726	October 2020 N=726	January 2021 N=728	March 2021 N=735	May 2021 N= 723
Wash hands with water only	30.73±38.09 [b]	29.36±37.82 [bc]	31.43±37.48 [b]	34.34±39.03[a] [bc]	33.55±38.60 [abc]	36.79±39.68 [ab]	37.58±39.03[a]	35.48±38.87 [abc]	35.50±38.24 [abc]
Wash hands with soap	90.39±18.22 [ab]	90.65±18.06 [a]	88.44±20.13 [abc]	89.06±20.15 [abc]	88.67±20.55 [abc]	88.92±19.81 [abc]	86.38±22.33[cd]	87.21±21.28[bc] [d]	83.94±26.44 [d]
Use hand sanitizer	70.80±30.70 [c]	71.64±31.55 [abc]	74.18±29.69 [abc]	75.72±29.49 [abc]	76.17±29.20[a]	75.78±28.56 [ab]	74.44±29.69 [abc]	75.40±29.57 [abc]	70.91±32.38 [b]

Different superscripts indicate significant differences between months

Table S7. Levels of produce washing during the COVID-19 Pandemic (April 2020-May 2021) [0= Never, 50= Sometimes, 100 = Always]

	**April 2020 N= 703**	**May 2020 N=732**	**June 2020 N=707**	**July 2020 N=716**	**August 2020 N= 726**	**October 2020 N=726**	**January 2021 N=728**	**March 2021 N=735**	**May 2021 N= 723**
Wash fruits and vegetables with water	81.56±28.94 [a]	82.37±29.24 [a]	81.36±28.08 [a]	81.45±28.54 [a]	82.61±27.62 [a]	81.18±28.02 [a]	79.74±28.70 [ab]	81.02±28.22 [a]	76.00±32.12 [b]
Wash fruits and vegetables with soap	40.26±40.88 [a]	34.70±40.60 [a]	36.09±39.90 [a]	37.81±40.08 [a]	37.44±40.03 [a]	39.14±40.25 [a]	38.83±39.74 [a]	35.45±39.79 [a]	36.49±39.40 [a]

Different superscripts indicate significant differences between months
